# Degenrative Fibroid and Sclerosing Peritonitis

**DOI:** 10.1155/2012/218126

**Published:** 2012-08-13

**Authors:** Michael Critchley, John Bagley, Pervis Iqbal

**Affiliations:** Department of Obstetrics and Gynaecology, Doncaster Royal Infirmary Hospital, Armthorpe Road, Doncaster DN2 5LT, UK

## Abstract

Sclerosing peritonitis is a rare condition characterised by ascites, peritoneal and bowel wall thickening. Causes reported in the literature include luteal ovarian the comas, peritoneal dialysis, peritoneal chemotherapy and liver cirrhosis. We report an interesting case of a woman presenting with diarrhoea, abdominal distension, ascites and pleural effusion. She was subsequently diagnosed with Sclerosing Peritonitis caused by a degenerating fibroid which was successfully treated by Total Abdominal Hysterectomy and Bilateral Salpingoophrectomy.

## 1. Introduction

Sclerosing peritonitis is a rare condition characterised by ascites, peritoneal, and bowel wall thickening. Causes reported in the literature include luteal ovarian thecomas [1], peritoneal dialysis, peritoneal chemotherapy, and liver cirrhosis [2]. 

We report an interesting case of a woman presenting with diarrhoea, abdominal distension, ascites, and pleural effusion. She was subsequently diagnosed with sclerosing peritonitis caused by a degenerating fibroid which was successfully treated by total abdominal hysterectomy and bilateral salpingo-ophrectomy. 

## 2. Case Report

A 66-year-old lady presented to the general surgeons with a history of diarrhoea and abdominal distension. Examination revealed ascites and a CT scan was organised; this showed gross ascites with small pleural effusions, bulky ovaries, a fibroid uterus, and multiple peritoneal and mesenteric deposits. Tumour makers were within normal limits with Ca 125 17, CEA 2.1, and Ca19 9 6.2. A gynaecological oncology opinion was sought and an ascitic drain sited for symptom relief and to obtain possible cytological diagnosis. Histological analysis of ascitic fluid on two separate occasions revealed leukocytes, mesothelial cells, and scattered histiocytes with no malignant cells present. Further immunohistochemistry was performed confirming the cells to be lymphoid in origin.

She continued to be symptomatic with abdominal distension and vomiting and therefore, a diagnostic laparoscopy was performed by the general surgeons. Laparoscopy confirmed gross ascites with what appeared to be widespread carcinomatosis with peritoneal and omental seedlings, bulky uterus and ovaries. Omental biopsies that were taken revealed an inflammatory process suggestive of possible sclerosing peritonitis. Histology showed omental fat with fibrous proliferation at the surface infiltrated by inflammatory cells and nodules of mesothelial proliferation, with no evidence of neoplastic infiltration. Immunohistochemical analysis was negative. 

Her clinical condition remained unchanged, and she required further ascitic drains for symptom relief. An MRI scan confirmed CT findings, and her case was discussed in detail at the Gynaecology and Colorectal MDT. 

As extensive investigation failed to establish a definitive diagnosis. In view of the small possibility of the fibroid uterus being responsible for her condition the decision was taken to perform a total abdominal hysterectomy, bilateral Salpingo-ophrectomy, omental biopsy, and serosal bowel biopsy. The gynaecological procedure was completed without difficulty with note only of an enlarged fibroid uterus. The peritoneal cavity was generally fibrosed extending throughout the bowel surface. Macroscopically the bowel was firm, solid with no obvious peristalsis due to the serosal fibrosis explaining the symptoms of obstruction. The bowel was inspected by the bowel surgeon and a number of biopsies were taken. 

Histological analysis from the peritoneal fluid showed mixed inflammatory cells and markedly reactive mesothelial cells. Interestingly, the ovaries were normal and the uterus contained an infracted ischaemic fibroid. Sections from the omentum and small bowel showed a fibrous process with spindle cells and inflammation. Overall this indicated diffuse sclerosing peritonitis. 

Her postoperative recovery was uneventful and at her six-week follow-up she had made an excellent recovery. She remained asymptomatic at her six-month follow-up. A CT that was performed at the time when compared to previous imaging showed complete resolution of her ascites and a normal appearance of the bowel. 

## 3. Discussion

Sclerosing peritonitis is a rare inflammatory process which is often related to luteinised ovarian thecoma, peritoneal dialysis, chemotherapy, and liver cirrhosis [1, 2]. 

Clinical presentation much like ovarian carcinoma is nonspecific including abdominal pain, weight loss, diarrhoea, and abdominal distension [3]. Subsequently, complications may arise from ascites or small bowel obstruction. Ovarian masses may also be present but are usually caused by benign ovarian thecomas. CA125 level is invariably normal in most cases. Macroscopic findings are of ascites, peritoneal, and small bowel thickening [4].

The process of peritonitis consists of fibroblast and myofibroblastic cell proliferation separated by collagen, fibrin, and inflammatory cells.  Ovarian spindle cells are commonly demonstrated [5]. 

A treatment regime for sclerosing peritonitis has not been established but there are case reports in the literature that have indicated a role for total abdominal hysterectomy and BSO in removal of the thickened peritoneum and partial resection of the small bowel. 

Our literature search has failed to establish any previous reported cases of a fibroid uterus associated with sclerosing peritonitis. 

We reported a case of diffuse idiopathic sclerosing peritonitis. This is a rare benign pathological condition and in contrast to the majority of case reports where an ovarian mass was a feature, which was absent in our case. After a prolonged hospital admission the patient was treated with TAH and BSO which has subsequently led to a full and uneventful recovery.

## 4. Conclusion

In unidentified causes of sclerosing peritonitis with a fibroid uterus this should be considered as a potential cause for the condition. Total abdominal hysterectomy and bilateral salpingo-ophrectomy should be considered as a possible treatment for the condition if their family is completed. In patients who have not completed their family a myomectomy could be considered.
